# An audit of the screen-and-treat intervention to reduce cryptococcal meningitis in HIV-positive patients with low CD4 count

**DOI:** 10.4102/phcfm.v10i1.1779

**Published:** 2018-08-08

**Authors:** Egide Ndayishimiye, Andrew J. Ross

**Affiliations:** 1 Health, College of Health Sciences, University of KwaZulu-Natal, Prince Mshiyeni Memorial Hospital, South Africa; 2 Department of Family Medicine, School of Nursing and Public Health, College of Health Sciences, University of KwaZulu-Natal, South Africa

## Abstract

**Background:**

HIV-associated cryptococcal meningitis (CCM) and related mortality may be prevented by the effective implementation of a screen-and-treat intervention.

**Aim:**

The aim of this study was to assess the effectiveness of the screen-and-treat intervention at a regional hospital in KwaZulu-Natal province, South Africa.

**Method:**

This was a descriptive study in which the records of patients seen in 2015 and 2016 with a CD4 count ≤ 100 cell/mm^3^ were retrieved from National Health Laboratory Service (NHLS) records and matched against patients admitted for HIV-associated CCM.

**Results:**

A total of 5.1% (190 out of 3702) patients with CD4 count ≤ 100 cell/mm^3^ were cryptococcal antigen positive (CrAg +ve), of whom 22.6% (43 out of 190) were admitted with CCM. Patients who were CrAg +ve had significantly lower CD4 counts (mean CD4 = 38.9 ± 28.5) when compared to CrAg –ve patients (mean CD4 = 49.9 ± 37.4) with *p* = 0.0001. Only 2.6% (5 out of 190) of patients were referred for a lumbar puncture (LP) as part of the screen-and-treat intervention, whilst 38 who were CrAg +ve self-presented with CCM. Eighty-eight patients were admitted for suspected CCM: eight because of the screen-and-treat-intervention (none of whom had meningitis based on cerebrospinal fluid results) and 80 of whom self-presented and had confirmed CCM. The overall mortality of patients admitted with CCM was 30% (24 out of 80).

**Conclusion:**

The current ad-hoc screen-and-treat intervention was ineffective in detecting patients at risk of developing CCM. Systems need to be put in place to ensure that all CrAg +ve patients have an LP to detect subclinical CCM to improve the outcome for those with HIV-associated CCM.

## Introduction

Cryptococcus meningitis is caused by *Cryptococcus neoformans*, an encapsulated yeast-like organism ubiquitous in the environment.^1,2^ Although exposed to *C. neoformans* pathogens, most people with a normal immune system do not acquire this infection. However, those with a lowered immune response are susceptible to cryptococcal infection, which has a particular tropism for the central nervous system, frequently causing fatal cryptococcal meningitis (CCM).^3^ This is a common opportunistic infection in Southeast Asia and sub-Saharan Africa,^1^ and is an Acquired Immune Deficiency Syndrome (AIDS)-defining illness in patients with late-stage HIV infection. It remains a major cause of HIV-related mortality worldwide with the largest burden of the disease being in sub-Saharan Africa, where health care access is often limited.^4,5,6^

South Africa (SA) has the largest HIV epidemic in the world, with approximately 7.06 million South Africans living with the virus in 2017 (12.6% of the total South African population).^7^ Since 2004, the antiretroviral therapy (ART) programme in SA has grown considerably, and by 2016, it was estimated that 3 929 000 (56%) people living with HIV (PLWHIV) were on treatment.^8,9^ In 2015, SA adopted the United Nations AIDS 90–90–90 ambitious but achievable treatment targets to help end the AIDS epidemic by 2020. In this programme, it is expected that 90% of the South African population will be aware of their HIV status; 90% of those who test positive will be on ART, and 90% of those who are on ARTs will be virally supressed by 2020.

Despite these ambitious goals and the increased coverage of ART in SA, the incidence of HIV-associated CCM remains high with a case-fatality rate ranging between 30% and 50%.^2,4,10,11^ There is a high susceptibility to cryptococcal infection and the subsequent development of CCM among patients with a CD4 count ≤ 100 cells/mm^3^ as well as a high associated mortality.^12,13^ Therefore, in 2011, the World Health Organization (WHO) recommended that prior to initiating ART in populations with a high prevalence of cryptococcal (Cr) antigenaemia, all ART-naïve adults with a CD4 count ≤ 100 cells/mm^3^ should be routinely screened. People with evidence of cryptococcal disease should then be assessed for evidence of CCM and treated when positively diagnosed.^14,15^

In 2014, the South African Department of Health adopted the WHO recommendations,^14^ and a screen-and-treat intervention was added to the South African HIV guidelines^16^ which require that all HIV-positive patients with a CD4 ≤ 100 cells/mm^3^ be screened for Cr antigenaemia. All those who are Cr antigen positive (CrAg +ve) should be assessed for CCM, and those with symptoms suggestive of CCM should have a lumbar puncture (LP) and be treated if the diagnosis is confirmed. The purpose of this proactive screen-and-treat intervention is to reduce CCM-related deaths.^10,16^

The aim of this study was to assess the effectiveness of the current screen-and-treat intervention for HIV-positive patients with a CD4 count ≤ 100 cells/mm^3^ who presented for care either at a large regional hospital in Durban or at a clinic that referred patients to the hospital, during 2015 and 2016.

## Methods

Prince Mshiyeni Memorial Hospital (PMMH) is a 1200-bed regional hospital situated on the outskirts of Umlazi Township, Durban, and serves a population of approximately 2 million people with an estimated HIV prevalence of 16.9%.^17^ There are 17 clinics in the hospital ‘catchment area’ that refer the patients to the hospital. All laboratory investigations (bloods, cerebrospinal fluid [CSF], etc.) from these clinics and PMMH hospital are sent to the National Health Laboratory Service (NHLS) laboratory based at the hospital for analysis. In accordance with the National HIV guidelines, all HIV-positive patients presenting to any of the 17 clinics or to PMMH must have a baseline CD4 count, and those specimens where the CD4 count is ≤ 100 cells/mm^3^ are reflexively screened for the presence of CrAg using the same blood specimen. Those patients who are CrAg +ve are assessed by the clinicians for symptoms suggestive of CCM (headache, photophobia and/or confusion), and if symptomatic, are offered an LP to rule it out as part of the screen-and-treat intervention.

This study was divided into two parts, the first being a review of the data from the NHLS laboratory at PMMH of all those with a CD4 count ≤ 100 cells/mm^3^. This was followed by the second part, which was a review of the hospital records of all patients referred for an LP for suspected CCM and all those admitted to the hospital with confirmed CCM.

### Part A: National Health Laboratory Service data review

The data for all HIV-positive patients older than 12 years of age seen at PMMH or the clinics who had a CD4 count ≤ 100 cells/mm^3^ between June 2015 and May 2016 were accessed from the NHLS laboratory records. These records were reviewed to determine the prevalence of Cr antigenaemia, and to match the data from the screened population against that of patients admitted to PMMH for an LP or CCM management using name, surname outpatient (OP) and/or inpatient number, age and date of admission.

### Part B: Hospital records

All of the ward admission books from all of the adult medical wards at PMMH were reviewed by the principal investigator. The records of all patients (≥ 12 years) admitted for suspected meningitis were retrieved from the hospital records and reviewed in detail. Only the files of those patients who had an LP for suspected symptomatic CCM, and those with a confirmed diagnosis based on the CSF results between June 2015 and May 2016, were included in the study. A standardised data collection sheet was used to record age, gender, weight, symptoms of meningitis, CD4 count, ARTs duration, previous conditions, symptomatic presentation or screening, CSF results, Amphotericin B duration, complications, outcome (death, discharge) and length of stay (recorded in days). The records were reviewed carefully to establish if patients were referred to the hospital because of the screening process or whether they presented to the hospital because of symptoms suggestive of CCM (self-presented to the hospital). Data were entered using a Microsoft Excel Software package and analysed descriptively using the IBM SPSS Statistical Software, version 25. The Fisher’s exact test was used for categorical data and the independent sample *T* test was used for the numerical data with the level of significance being set at 0.05.

## Ethical Considerations

Ethical approval for the study was granted by Biomedical Research Ethic Committee (BREC REF No: BE402/16) of the University of KwaZulu-Natal (UKZN). Permission to conduct the study was granted by Prince Mshiyeni Memorial Hospital (PMMH) management and National Health Laboratory Service (NHLS) management.

## Results

### Part A: National Health Laboratory Service data review

Based on the retrieved NHLS laboratory results, 3702 patients aged between 12 and 86 years with a CD4 count ≤ 100 cell/mm^3^ were seen at the clinics or hospital between June 2015 and May 2016. The mean age of these patients was 35 ± 9.8 years, male patients represented 49.9% (1847 out of 3702) of the population and the mean age of male patients was older (mean age 36 ± 9.31 years) than female patients (mean age 33 ± 10.1 years) with *p* = 0.001. The CD4 counts ranged between 1 and 100 cells/mm^3^ (mean = 48.3 ± 28.7), with male patients having a lower mean CD4 count (mean CD4 = 47 ± 28.4) than female patients (mean CD4 = 50 ± 28.9) with *p* = 0.003, a summary of those who were CrAg +ve and –ve being presented in Table 1. Patients who were CrAg +ve had significantly lower CD4 counts (mean CD4 = 38.9, SD 28.5) compared to the CrAg –ve patients (mean CD4 = 49.9, SD 37.4) with *p* = 0.0001.

**TABLE 1:** Summary of the CD4 count and viral load of patients with a CD4 count ≤ 100.

	Number	Male patients		Female patients		Mean CD4 count		Mean viral load	
		*n*	%	*n*	%	cells/mm^3^	*p*-value	copies/mL	*p*-value
Antigen +ve	190	115	60.5	75	39.5	38 ± 28.5	-	195 182 ± 341 002	-
Antigen −ve	3512	1731	49.3	1781	50.7	49 ± 37.4	-	207 126 ± 597 814	-
**Total**	**3702**	**1847**	**49.9**	**1856**	**50.1**	**-**	**< 0.0001**	**-**	**0.879**

**FIGURE 1:**
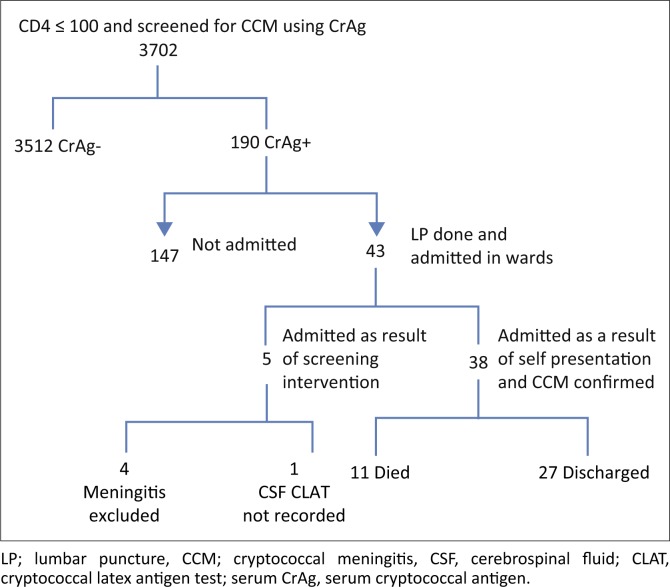
Flow diagram of patients with CD4 count ≤ 100 cells/mm^3^ who were screened for cryptococcal meningitis.

Figure 1 shows the flow of patients with a CD4 count ≤ 100 cells/mm^3^ who were screened for CrAg. In total, 3702 patients had a CD4 count ≤ 100 cells/mm^3^, of whom 3512 (94.9%) were CrAg –ve, and 190 (5.1%) were CrAg +ve, of whom 43 (22.6%) were admitted either as a result of screening or who self-presented with symptoms of CCM.

**FIGURE 2:**
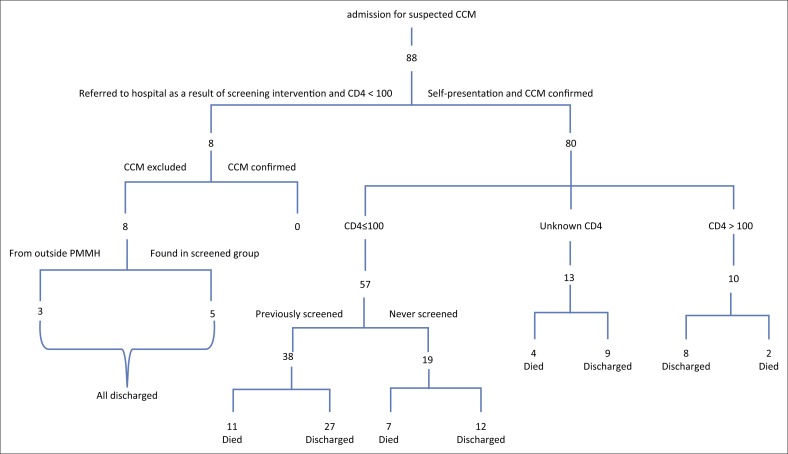
Flow diagram of patients admitted to Prince Mshiyeni Memorial Hospital for suspected cryptococcal meningitis.

Based on the data collected from the NHLS at PMMH, only five patients (5 out of 43; 11.6%) with a CD 4 ≤ 100 cells/mm^3^ had evidence of an LP performed at the hospital as part of screen-and-treat intervention, none of whom were diagnosed with CCM. The remaining 38 (88.4%) patients who had an LP all self-presented to PMMH hospital with symptoms of meningitis (Table 2), and all had LP evidence of CCM based on CSF Cryptococcal latex antigen test (CLAT) and/or culture results. These patients are included in the 80 patients who self-presented to the hospital (Figure 2). Although the laboratory and hospital records were extensively reviewed, no evidence could be found to indicate that any of the other 147 (77.4%) CrAg +ve patients had an LP or had been assessed for meningitis.

**TABLE 2:** Patients with CD4 ≤ 100 whose cryptococcal antigen was positive and who were admitted to Prince Mshiyeni Memorial Hospital where a lumbar puncture was performed (*n* = 43).

	Admitted as a result of screened (*n* = 5)			Self-presented with symptoms suggestive of CCM (*n* = 38)			
	*n*	%	mean	*n*	%	mean	
Female	1	20	-	14	36.8	-	0.643
Male	4	80	-	24	63.2	-	0.643
Age	-	-	35 ± 10		34 ± 6	-	0.796
CD4 count (cells/mm^3^)	16 ± 16	-	-	38 ± 25		-	0.043
Viral load (copies/mL)	181 025 ± 37 232	-	-	130 696 ± 13 6003		-	0.301

CCM, cryptococcal meningitis.

### Part B: Review of hospital records

Between June 2015 and May 2016, 80 patients were admitted to PMMH (this includes the 38 patients in Figure 1 who self-presented) and treated for HIV-associated CCM of whom 57 (71.3%) had a CD4 count ≤ 100 cells/mm^3^, 10 patients had a CD4 count > 100 cells/mm^3^ and 13 did not have their CD4 count recorded (Figure 3 and Table 3). Most patients who were admitted complained of headache (88.6%), photophobia (60%) and/or confusion 30.7%.

**TABLE 3:** Summary of the patients admitted with confirmed cryptococcal meningitis (*N* = 80).

CD4 count	No.	%	Male patients		Female patients		Age in years		CD4 mean cell/mm^3^
			*n*	%	*n*	%	Mean	Range	
≤ 100 cells/mm^3^	57	71.2	35	67.3	22	78.6	34 ± 6.875	21–52	37 ± 27.4
Unknown	13	16.3	11	21.2	2	7.1	35 ± 7.263	24–47	-
>100 cells/mm^3^	10	12.5	6	11.5	4	14.3	38 ± 5.959	30–49	155.1 ± 74.2
**Total**	**80**		**52**	**-**	**28**	**-**	**35 ± 6.911**	**21–52**	**55 ± 56.484**

No, number

The mean age of patients admitted with CCM was 34.6 ± 6.9 years (range 21–52 years). All patients with CSF evidence of CCM were treated with Amphotericin B (0.7 mg/kg–1 mg/kg) for two weeks together with a high dose of fluconazole. A plan for initiating ARTs four weeks post- discharge was made if patients were not already on ARTs. Besides the 80 patients who had confirmed CCM on CSF, eight were admitted as a result of the screen-and-treat intervention who had LPs. However, only five of these appeared in the PMMH NHLS CrAg results record, and it is possibly that the other three came from outside the PMMH catchment area. None of the screened patients had confirmed CCM on LP.

In total, 30% (24 out of 80) of patients admitted with CCM died, of those whose CD4 was ≤ 100 cell/mm^3^, 31.6% (18 out of 57) died, whilst of those with a CD4 > 100 cell/mm^3^, 20% (2 out of 10) died, as did 30.8% (4 out of 13) of those whose CD4 count was unknown. On average, the patients who died had a lower CD4 count (mean CD4 count = 40.35 cell/mm^3^ ± 36.5 cell/mm^3^) than those who were discharged (mean CD4 count = 54.0 cell/mm^3^ ± 60.1 cell/mm^3^) with *p* = 0.237 (see Table 4).

**TABLE 4:** Outcome profile of patients admitted with cryptococcal meningitis (*N* = 80).

CD4 count (cells/mm^3^)	Profile of patients who died (*n* = 24)			Profile of patients who were discharged (*n* = 56)		
	Number	%	Mean CD4 count	Number	%	Mean CD4 count
≤ 100	18	31.6	31.72 ± 26.437	39	68.4	37.0 ± 27.4
>100	2	20	118 ± 8.4	8	80	155.1 ± 74.2
Unknown	4	30.8	-	9	69.2	-
**Total**	**24**	**30**	**40.35 ± 36.5**	**56**	**70**	**54.0 ± 60.1**

## Discussion

In the NHLS component of the study, 3702 patients presented for care at PMMH and the 17 clinics between June 2015 and May 2016 with a CD4 ≤ 100 cells/mm^3^. Although progress is being made towards achieving the 90–90–90 targets, many patients continue to present late in the progression of the disease. The findings of a recent study showed that 85.5% of HIV-positive South Africans were aware of their status,^9^ and as ARTs have been widely available since 2004, it is a concern that so many patients continue to present only when their HIV is at an advanced stage. These findings suggest that despite extensive publicity to encourage people to test early and regularly for HIV and to know their status, many only become aware of their HIV status when they develop symptoms, by which time their CD4 counts are likely to be very low. Since the early 1990s, considerable resources have gone into HIV campaigns encouraging safe sexual behaviour and the importance of knowing one’s status. However, it would appear that a more nuanced understanding of the factors influencing when people choose to test for HIV and wish to start treatment is needed if all members of the population are to be encouraged to test early to know their HIV status and access ART.

In this study, only 5.1% (190 out of 3702) of the patients with a CD4 count of ≤ 100 cell/mm^3^ had Cr antigenaemia. These findings are higher than the 2.1% with Cr antigenaemia reported from a study in Soweto,^18^ consistent with studies in SA and UK,^19,20,21^ lower than the 13% reported from a 2009 study in Cape Town,^2^ and much lower than the 19% found in Uganda^22^ and the 17.7% reported in Cambodia.^23^ With *C. neoformans* being ubiquitous in the environment, it is not clear why the percentage of patients with Cr antigenaemia varies widely between patients with a low CD4 count, and may have to do with the incidence of cryptococcal disease in the population being studied.^18^

Jarvis et al. reported that a negative CrAg was a 100% negative predictor for the development of CCM in patients with a low CD4 count.^2^ This is helpful for clinicians, as it means that among the 3512 patients who were CrAg –ve in this study, there was no need to provide primary prophylaxis against CCM.^2,11,15^ However, based on a cohort of patients tested in 2011, Govender et al. reported that a small number of patients (7 out of 1430; 0.5%) developed CCM despite their initial CrAg being negative,^18^ as did Pongsa who reported that 0.8% (1 out of 119) of patients developed CCM despite having an initial CrAg that was negative.^24^ Govender concluded that these patients with very low CD4 counts developed CCM because of the delay in initiation of ARTs. The current South African guidelines recommend that all patients who are CrAg –ve do not need primary prophylaxis with fluconazole, but need to be fast-tracked onto ARTs as the subsequent improvement in the CD4 count is the best defence against CCM.^15,24^

Cryptococcal antigen immunoassays have been shown to be effective in identifying patients at risk of developing subclinical cryptococcal infection before ART initiation.^2,25^ Despite this, there is currently no consensus about how to manage patients who are CrAg +ve. The current South African HIV guidelines recommend that only those patients with symptoms suggestive of CCM should have an LP to confirm the diagnosis, and that those who are asymptomatic should receive high-dose fluconazole to prevent its development.^15,24^ Whilst a 2005 Cochrane review reported a reduction in the incidence of cryptococcal disease, there was no reduction in mortality associated with CCM from such approach.^26^

In this study, laboratory records show that only 2.6% (5 out of 190) of patients who were CrAg +ve had an LP at PMMH as part of the screening programme. In addition, no patient identified for an LP in this manner was diagnosed with CCM on LP, which suggests that clinicians were not effective in identifying patients with possible early onset of CCM. These results are very different from the findings from Gauteng province, where 41% (99 out of 244) of patients who were CrAg +ve were symptomatic, of whom 56 (57%) had an LP and 59% (33 out of 56) were diagnosed with CCM.^27^ Furthermore, the study in Gauteng reported that 26% (8 out of 31) of the asymptomatic CrAg +ve patients had CCM on LP.^27^ Further studies are needed to understand the discrepancy between these findings, which may be because of the large patient load in Umlazi, the ad-hoc manner in which patients who are CrAg +ve are assessed for symptoms of CCM, or the challenges of referring patients from the clinics to the hospital. Additional research is needed to determine how to effectively screen patients with low CD4 counts for symptoms of CCM.

Alternative approaches to managing patients who are CrAg +ve include providing CrAg titres to help clinicians decide which patients require an LP,^2,24^ or an LP for all patients who are CrAg +ve to exclude CCM, regardless of whether or not they are symptomatic.^25^ In a study in Thailand, where all patients who were CrAg +ve had an LP, 25% (3 out of 12) of patients had active CCM,^24^ whilst a retrospective review of patients who developed CCM in Cape Town showed that 28% (13 out of 46) who were CrAg +ve developed CCM.^2^ A follow-up study in Gauteng in 2012–2014, among HIV patients with a CD4 count ≤ 100 cells/mm^3^ who were CrAg +ve, showed that 25% had subclinical CCM.^27^

Based on the review of hospital records, 20% (38 out of 190) of patients who had been identified as CrAg +ve presented to the hospital with symptoms of CCM had their diagnosis confirmed LP. This finding is consistent with the study in Thailand,^24^ but much lower than the 66% – 71% reported in earlier studies among patients with stage IV HIV disease.^28,29^ These 38 patients may represent those patients who were lost to follow-up, which is not an uncommon occurrence,^18^ or be a missed opportunity for early identification of CCM and initiation of treatment prior to the onset of obvious symptoms of CCM. In this study, the clinicians appeared to be unable, in the screening process, to identify those patients who went on to develop CCM. This suggests that patients with low CD4 counts who are CrAg +ve and who develop CCM either have no or very subtle symptoms that are not recognised as possible precursors to the development of CCM. However, there may be patients who go on to develop CCM once they have started on ARTs, as was the experience in the study in Cape Town.^2,25^ More research is needed to understand the early symptoms that might be predictive of CCM.

Cryptococcal meningitis continues to have a significant mortality, despite the introduction of high-dose fluconazole into the South African treatment guidelines. Based on the data available at PMMH, the overall mortality in this study was 30%, which is consistent with other studies.^6,11,18^ It is important to note that the mortality was significantly higher in the cohort with a CD4 count ≤ 100 cell/mm^3^ (31.6%) and in the unknown CD4 cohort (30.8%) than in those with a CD4 count > 100 cell/mm^3^ (20%), supporting the call to identify patients who are HIV-positive and to initiate ART before they develop profound immunological failure. This was not an unexpected finding, as many studies have linked CCM-related mortality to a lower CD4 count.^2,19,20,30^

In the light of the high mortality associated with CCM, the challenges associated with early identification of subclinical CCM, the cost effectiveness of the screen-and-treat intervention (estimated to be cost-effective at a CrAg threshold of 0.6%),^18,20,30^ the high pickup rate of CCM in asymptomatic CrAg +ve patients,^24,27^ as well as the resources available in SA, serious consideration should be given to recommending LPs for all patients with a low CD4 count who are CrAg +ve.

Although this study is limited by the small numbers, with 20% mortality in those patients whose CD4 count ranged between 101 and 150 cells/mm^3^, consideration should include increasing the CD4 count level to 150 cells/mm^3^ for patients who are screened for CrAg. In 2015, Govender et al. reported a 97% yield of incident antigenaemia when the CD4 threshold was increased from 100 cells/mm^3^ to 150 cells/mm,^3,18^ suggesting that this increase in the CD4 cut-off threshold may help to timely detect CCM in this group of patients. Further study is needed in this area as well as assessing the effect of giving patients who are CrAg +ve prophylactic fluconazole, in keeping with the current guidelines, to determine what impact this would have on the development of CCM.

## Limitations

Because of the design of this study, it was not possible to follow up with patients who had *Cryptococcus antigenaemia*. Only a few patients were admitted for an LP following the screen-and-treat intervention, and the results need to be treated with caution. It is possible that some patients were referred but never presented to PMMH for LP and that some symptomatic patients were seen and managed at other health institutions, although the referral system is designed so that clinics in the Umlazi area refer patients to PMMH.

## Conclusions

The current ad-hoc system at busy public sector clinics does not appear to be effective identifying and/or referring patients with subclinical symptoms of CCM nor in reducing its associated mortality. Although screening for *Cr antigenaemia* is considered to be cost-effective, the benefits are currently not being realised. This may be because of the challenges associated with identifying patients with latent or early CCM as well as the challenges within the health care system that include large numbers of patients, overloaded staff and the failure of patients to return for follow-up. Although this is a small descriptive study limited to one location, it does provide data based on day-to-day practice with caution being advised when interpreting the data. Based on the findings of this research, we recommend that a system be considered to ensure that all patients with *Cr antigenaemia* are offered an LP to exclude CCM, and that the cut-off point for assessing *Cr antigenaemia* be increased to a CD4 count of 150 cells/mm^3^.

It is also very important that all patients with a low CD4 count be fast-tracked onto ARTs so as to reduce the risk of developing CCM. Further research is needed to assess the impact of these recommendations, and the effectiveness of other interventions aimed at reducing the mortality from CCM. This includes the use of oral fluconazole in patients who have Cr antigenaemia but not CCM, and high-dose fluconazole in combination with Amphotericin B for the management of CCM.
